# Identification of the key genes associated with chemotherapy sensitivity in ovarian cancer patients

**DOI:** 10.1002/cam4.3122

**Published:** 2020-05-22

**Authors:** Hui Zheng, Meiqin Zhang, Shuang Ma, Wenting Yang, Suhong Xie, Yanchun Wang, Yixuan Liu, Jinyan Kai, Qian Ma, Renquan Lu, Lin Guo

**Affiliations:** ^1^ Department of Clinical Laboratory Fudan University Shanghai Cancer Center Shanghai China; ^2^ Department of Oncology Shanghai Medical College Fudan University Shanghai China; ^3^ Department of Gynecologic Oncology Fudan University Shanghai Cancer Center Shanghai China; ^4^ Genenexus Technology Corporation Shanghai China

**Keywords:** chemotherapy sensitivity, key genes, NGS, ovarian cancer, pathways

## Abstract

**Background:**

Ovarian cancer (OC) is the deadliest gynecological cancer. The absence of biomarkers in early detection and chemotherapy resistance is a principal cause of treatment failure in OC.

**Methods:**

In this study, next generation sequencing (NGS) was used to sequence the mRNA of 44 OC patients including 14 chemotherapy insensitive and 18 sensitive patients. Differentially expressed genes (DEGs) from OC patients (compared with healthy controls) and chemotherapy sensitive patients (compared with chemotherapy insensitive patients) were identified by edgeR v3.12.0 in R v3.2.2, which were enriched using Gene Ontology (GO) database and Kyoto Encyclopedia of Genes and Genomes pathway (KEGG). The common DEGs in cancer occurring and chemotherapy sensitivity were further screened. Among them, genes participating in chemotherapy sensitivity associated pathways were regarded as chemotherapy sensitivity‐related key genes. Quantitative real‐time PCR (qPCR) and immunohistochemistry (IHC) were used to verify the expression of the key genes.

**Results:**

We found 1588 DEGs between OC patients and healthy controls (HCs), which were mainly enriched in cell cycle pathway. Meanwhile, 249 DEGs were identified between chemotherapy sensitive and insensitive OC patients, which were mainly enriched in MAPK signaling pathway, ERBB signaling pathway, TNF signaling pathway, and IL‐17 signaling pathway. Thirty‐five DEGs were shared in chemotherapy sensitivity group and cancer occurring group. Among them, there are five genes (JUND, JUNB, MUC5B, NRG1, and NR4A1) participating in the above four chemotherapy sensitivity‐related pathways. It is remarkable that JUND is in the upstream of MUC5B in IL‐17 signaling pathway and their expressions were verified by qPCR and IHC.

**Conclusions:**

The expression levels of the key genes related to chemotherapy sensitivity might be used as biomarkers to predict the treatment outcome and as a target to improve prognosis.

## Graphical abstract

The expression levels of the key genes related to chemotherapy sensitivity might be used as biomarkers and targets to predict the chemotherapy effect and treatment of OC patients.

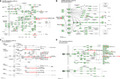

## 1 INTRODUCTION

Ovarian cancer (OC) is one of the most fatal tumors in women. The main treatment methods are surgery and chemotherapy with paclitaxel and platinum.^1^ However, about 25% of OC patients will be resistant to chemotherapy drugs within 6 months,^2^ even worse, most OC patients eventually develop severe drug resistance after long‐term treatment, resulting in high recurrence rate and poor prognosis.^3^ Therefore, it is urgent to uncover the molecular mechanism of chemotherapy resistance in OC.

Chemotherapy resistance in OC is mainly through reducing drug accumulation, increasing cellular detoxification, stimulating DNA repair, altering intrinsic apoptosis pathways, and regulating autophagy.^4^, ^5^, ^6^, ^7^, ^8^ These biological processes involve in multiple genes and multiple pathways. Gene mutation site,^9^, ^10^, ^11^, ^12^ mRNA expression level,^13^, ^14^, ^15^ transcription factor,^16^ miRNA,^17^, ^18^, ^19^ long non‐coding RNA,^20^, ^21^ epigenetic regulation^22^, ^23^ were widely studied to predict the chemotherapy response in OC. However, until now, there is no representative biomarker to predict the chemotherapy efficacy of OC in the clinic due to the complex mechanism of chemotherapy resistance coupled with the genetic heterogeneity of ovarian cancer patients. Thus, the key genes associate with chemotherapy sensitivity still need to be explored.

Here, we used next generation sequencing (NGS) combining bioinformatics technology to identify more chemotherapy sensitivity‐related key genes, which can be as targets to increase chemotherapy sensitivity.

## 2 MATERIALS AND METHODS

### 2.1 Clinical samples

Tissue samples from ovarian cancer patients without drug treatment were collected at Fudan University Shanghai Cancer Center from December 25, 2012 to August 31, 2017. Samples were stored at −80℃ until RNA isolation. The patients who recurred within 6 months after chemotherapy were regarded as chemotherapy insensitivity. Instead, the patients who did not recur over 6 months were regarded as chemotherapy sensitivity. A total of 44 patients (range, 30‐79 years old) were recruited including 14 chemotherapy insensitive and 18 sensitive patients. The pathological stage was defined according to UICC/AJCC and TNM classification system (https://www.uicc.org/resources/tnm), details were shown in Table 1. The research was authorized by the Ethics Committee of Fudan University Shanghai Cancer Center and informed consent was obtained from all patients.

**TABLE 1:** Histopathological characteristics of the ovarian cancer patients

Patient	Age (y)	UICC staging	PFS (mo)	Chemotherapy sensitivity
1	57	IIIC	No records	Insensitive
2	57	IIIC	36	Sensitive
3	59	IIIC	43	Sensitive
4	50	IIIC	4	Insensitive
5	57	IIIC	29	Sensitive
6	65	IIIC	61	Sensitive
7	55	IIIC	8	Insensitive
8	67	IV	3	Insensitive
9	58	IC	64	Sensitive
10	51	IV	17	Insensitive
11	72	IIIC	54	Sensitive
12	39	IIIC	27	Sensitive
13	57	IIIC	1	Indeterminacy
14	58	None	14	Sensitive
15	46	IV	13	Sensitive
16	42	IIIC	No records	Insensitive
17	64	IIIC	31	Sensitive
18	55	IIIC	4	Indeterminacy
19	45	IIIC	58	Indeterminacy
20	57	IIIC	13	Insensitive
21	58	IIIC	26	Sensitive
22	28	None	15	Sensitive
23	58	IIIC	16	Sensitive
24	53	IIIC	No records	No records
25	61	IV	11	Insensitive
26	79	None	No records	No records
27	52	IIIC	22	Sensitive
28	59	IIIC	9	Indeterminacy
29	50	IIIC	23	Sensitive
30	62	IV	20	Sensitive
31	64	IIIb	9	Indeterminacy
32	30	None	7	Insensitive
33	50	None	9	Indeterminacy
34	59	None	21	Sensitive
35	44	IIIC	23	Sensitive
36	50	None	13	Insensitive
37	49	III	8	Indeterminacy
38	30	IV	4	Insensitive
39	64	IIIC	9	Insensitive
40	48	None	9	Insensitive
41	51	IIIC	No records	No records
42	60	IIIC	4	Indeterminacy
43	56	IIIC	14	Insensitive
44	40	None	10	Indeterminacy

### 2.2 RNA isolation and sequencing

RNA was isolated from tumor tissues using TriReagent (Ambion Inc). Agarose gel electrophoresis was performed to determine the extent of RNA degradation and contamination. The purity of the RNA was also measured by Nanodrop (ND‐1000). The concentration was precisely quantified by Qubit. The integrity was assayed by Agilent 2100 and samples with a RIN value of 7 or above were used for further analysis. Small RNA sequencing libraries were created according to the IlluminaHTruSeq TM Small RNA Sample Preparation protocol. Segment sizes were selected by AMPure XP beads, and PCR enrichment was conducted to obtain the final cDNA library. HiSeq sequence was conducted after the library passed the inspection.

### 2.3 Identification of DEGs

The quality of RNAseq data was controlled by Fastp software. RNA‐seq data of OC tissues was blasted to the Hg19 human reference genome by STAR software. Quantification and standardization of genes used RSEM software (count FPKM TPM). The read count data were then analyzed to identify DEGs in OC tissues and normal tissues through edgeR v3.12.0 in R v3.2.2 with false positive discovery (FDR) correction. The read count data of normal tissues belong to the Common Fund's Genotype‐Tissue Expression (GTEX) (http://commonfund.nih.gov/GTEx/), the largest international normal tissue database. In addition, the read count data were also analyzed to identify DEGs in chemotherapy sensitive and insensitive tissues by the same method. The SVA software was used to eliminate the batch effect. The genes meeting the conditions of |log2 fold change (logFC)| >2 and *P* < .01 were considered as DEGs.

### 2.4 Enrichment analysis of DEGs

Gene Ontology (GO) database (http://www.geneontology.org/) and Kyoto Encyclopedia of Genes and Genomes pathway (KEGG) (http://www.kegg.jp) were used to enrich the DEGs in cancer occurring and chemotherapy sensitivity group separately. We utilized r packages “org.Hs.eg.db47” and “clusterProfile48” to identify the proteins belonging to corresponding pathway. *P* < .05 set as the threshold.

### 2.5 Real‐time PCR

RNA was extracted from tissues with ovarian cancer using DNA/RNA isolation kit (TIANGEN). Concentrations and purity of RNA were analyzed using a NanoDrop ND‐1000 Spectrophotometer. Up to 1 μg total RNA from each sample was reverse transcribed to cDNA using a PrimeScriptTM RT reagent kit (Takara). The following primer pairs were used to evaluate the relative expression level of each mRNA: β‐actin: F: 5′‐AAGGTGACAGCAGTCGGTT‐3′, R: 5′‐TGTGTGGACTTGGGAGAGG‐3′; JUND: F: 5′‐CAAGGACGAGCCACAGACG‐3′, R: 5′‐CCGTGTTCTGACTCTTGAGGG‐3′; MUC5B: F: 5′‐AACTGCACCGTGTACCTCTG‐3′, R: 5′‐TCGTGTTGATGCGGACTTGA‐3′. All primers were purchased from Sangon Biotech. Quantitative PCR assays were performed using Hieff UNICON Power qPCR SYBR Green Master Mix (YEASEN) and Applied Biosystems QuanStudio Dx Real‐Time PCR system. For each sample, 20 μL reactions were set up containing 10 μL 2× SYBR mix, 0.4 μL PCR forward primer (10 μM), 0.4 μL PCR reverse primer (10 μM), 2 μL template cDNA, and 7.2 μL RNase‐free water. All PCR reactions were performed in triplicate. The following cycling protocol was used: 95°C for 3 minutes, then 40 cycles with a two‐step programme (95°C for 10 seconds, 60°C for 30 seconds), and completed with a product dissociation cycle. The relative expression values for each target gene are shown as 2^−ΔCt^.

### 2.6 Immunohistochemical staining and evaluation

Formalin‐fixed and paraffin‐embedded sections were dewaxed in xylene and hydrated in grade alcohol, followed by inhibition of endogenous peroxidase activities with methanol containing 0.3% H_2_O_2_. After boiling in 10 mmol/L of citrate buffer (pH 6.0) for antigen retrieval and cooling down, the sections were blocked with 1% BSA and incubated overnight at 4°C with rabbit polyalonal antibody to JunD (Abcam, ab28837, 1:200) and MUC5B (Abcam, ab87376, 1:100). On the second day, these sections were incubated for another 45 minutes at 37°C. After washing with PBS, the sections were incubated with HRP‐conjugated secondary antibody (Shanghai Long Island Biotech) for 1 hour at room temperature, followed by reaction with diaminobenzidine, and counterstaining with hematoxylin.

## 3 RESULTS

### 3.1 OC‐related biological processes and pathways

**FIGURE 1:**
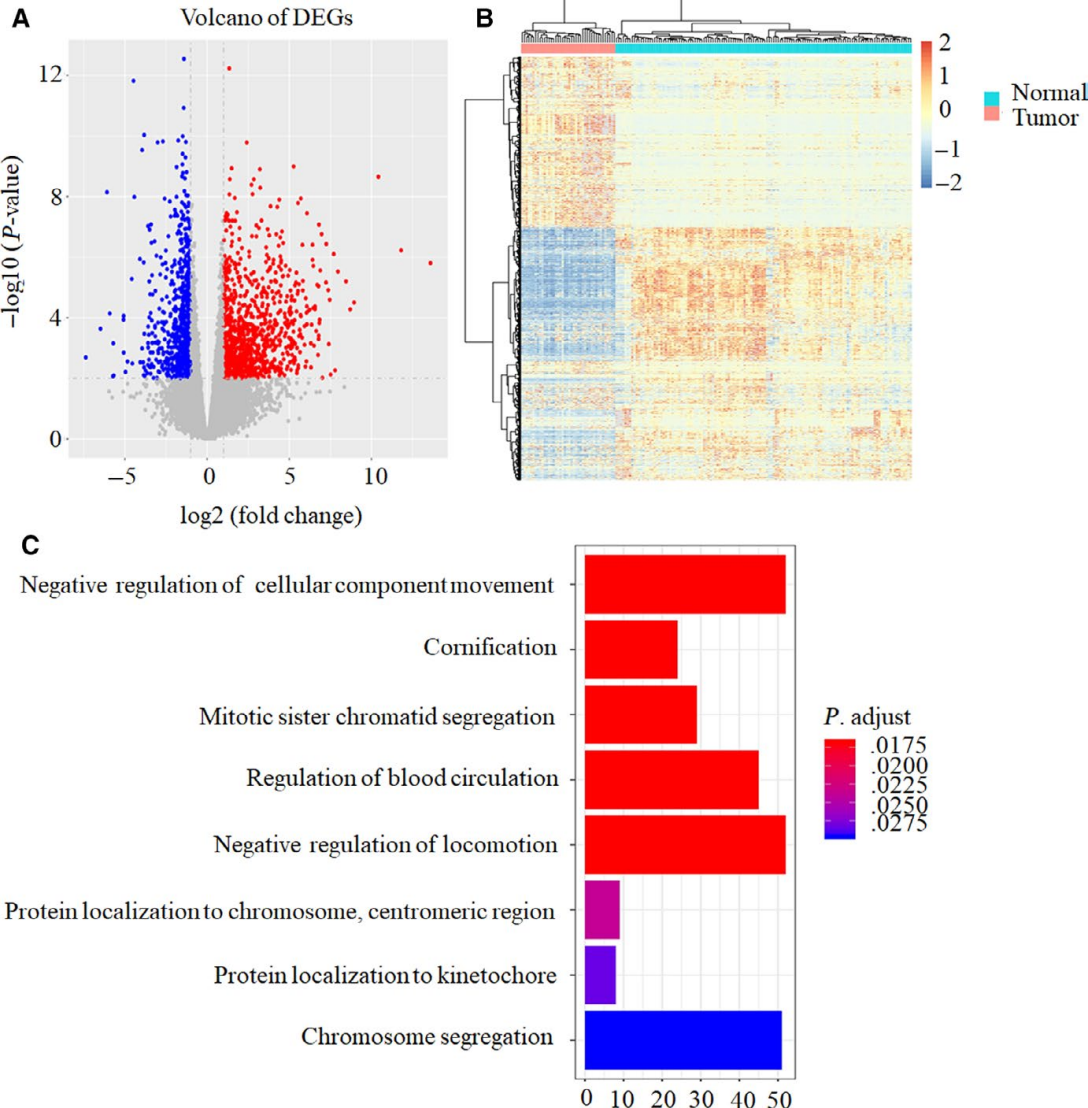
Identification of DEGs between OC and HCs and predicted their bio‐functions. (A) Volcano plots showed 945 up‐regulated genes (red) and 643 down‐regulated genes (blue) between 44 OC and 133 normal ovarian tissues (belonging to GTEX) using edgeR software with FDR <0.01 & |log2FC| >2. (B) Bidirectional hierarchical clustering of the total 1588 DEGs. (C) GO enrichment analyses of these DEGs. X‐axis represents the number of DEGs. Only the results with *P* < .05 were considered to be significant

A total of 1588 DEGs were identified between 44 OC and 133 healthy controls (HCs) (belonging to GTEX), including 945 up‐regulated genes and 643 down‐regulated genes in OC compared with HCs as shown in volcano plot (Figure 1A). The heat map successfully clustered the two groups of samples separately (Figure 1B).

GO analysis showed that several biological processes, such as cellular component movement, cornification, mitotic sister chromatid segregation, blood circulation, locomotion, protein localization, and chromosome segregation were associated with OC occurring (Figure 1C). KEGG results demonstrated that cell cycle pathway was related to OC occurring (Table 2).

**TABLE 2:** KEGG pathways analysis of DEGs between ovarian cancer and healthy controls

ID	Pathway	*P* value	Genes
hsa04110	Cell cycle	.000120948	E2F2, CDC14B, ORC1, TGFB2, CDC45, CDC6, ABL1, E2F1, MCM4, CCND3, TTK, CDC20, PKMYT1, CCNA1, CCNB1, ESPL1, BUB1B, CCNB2, PTTG1, PLK1, BUB1, CDK1, SFN

Taken together, the mechanism of OC occurring is complex involving multiple genes and biological processes.

### 3.2 Chemotherapy sensitivity‐related biological processes and pathways

**FIGURE 2:**
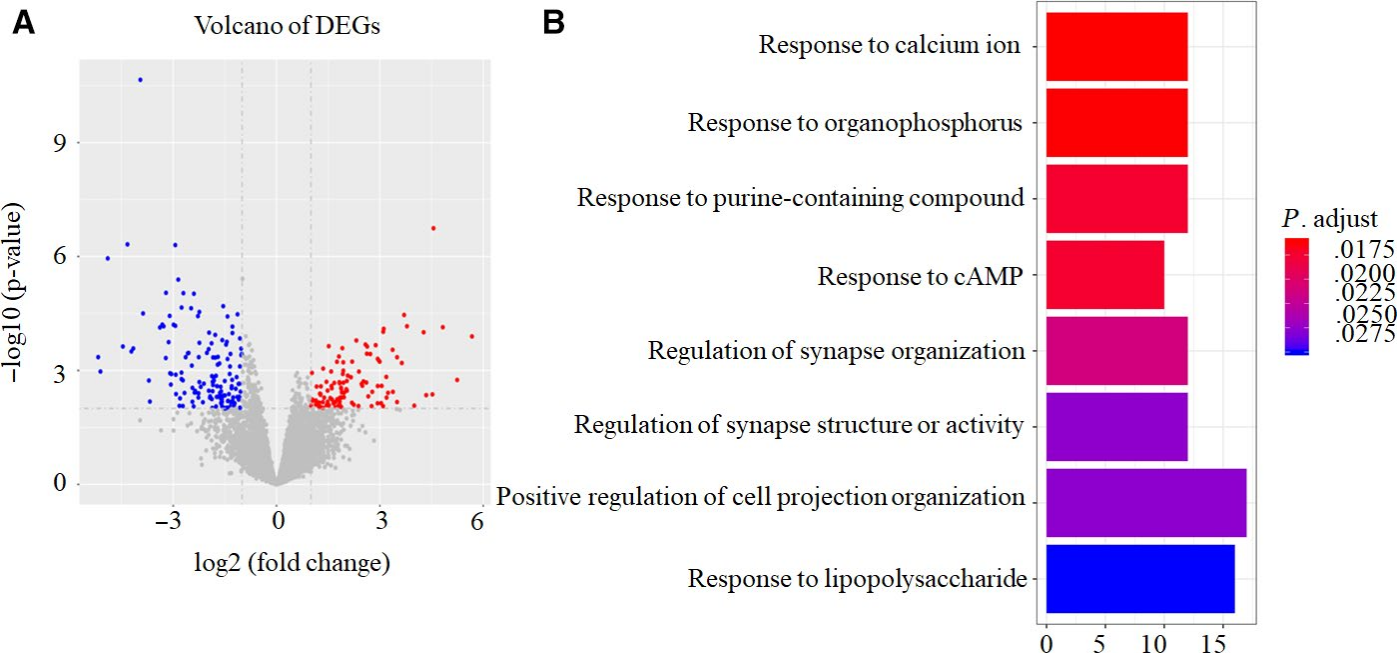
Identification of DEGs between chemotherapy sensitive OC tissues and chemotherapy insensitive OC tissues and predicted their bio‐functions. (A) 108 up‐regulated genes (red) and 141 down‐regulated genes (blue) were identified between 18 chemotherapy sensitive and 14 insensitive ovarian cancer patient tissues using edgeR software with FDR <0.01 & |log2FC| >2. (B) GO enrichment analyses of the total 249 DEGs. X‐axis represents the number of DEGs. Only the results with *P* < .05 were considered to be significant

A total of 249 DEGs were identified between 18 chemotherapy sensitive OC patients and 14 chemotherapy insensitive OC patients, 108 genes were up‐regulated and 141 genes were down‐regulated in chemotherapy sensitive OC patients compared with chemotherapy insensitive OC patients as shown in volcano plot (Figure 2A).

Furthermore, GO analysis demonstrated that DEGs mainly participated in the following biological processes: responding to calcium ion, organophosphorus, purine‐containing compound, cAMP, lipopolysaccharide, regulating of synapse organization, structure or activity, and positive regulating of cell projection organization (Figure 2B).

**FIGURE 3:**
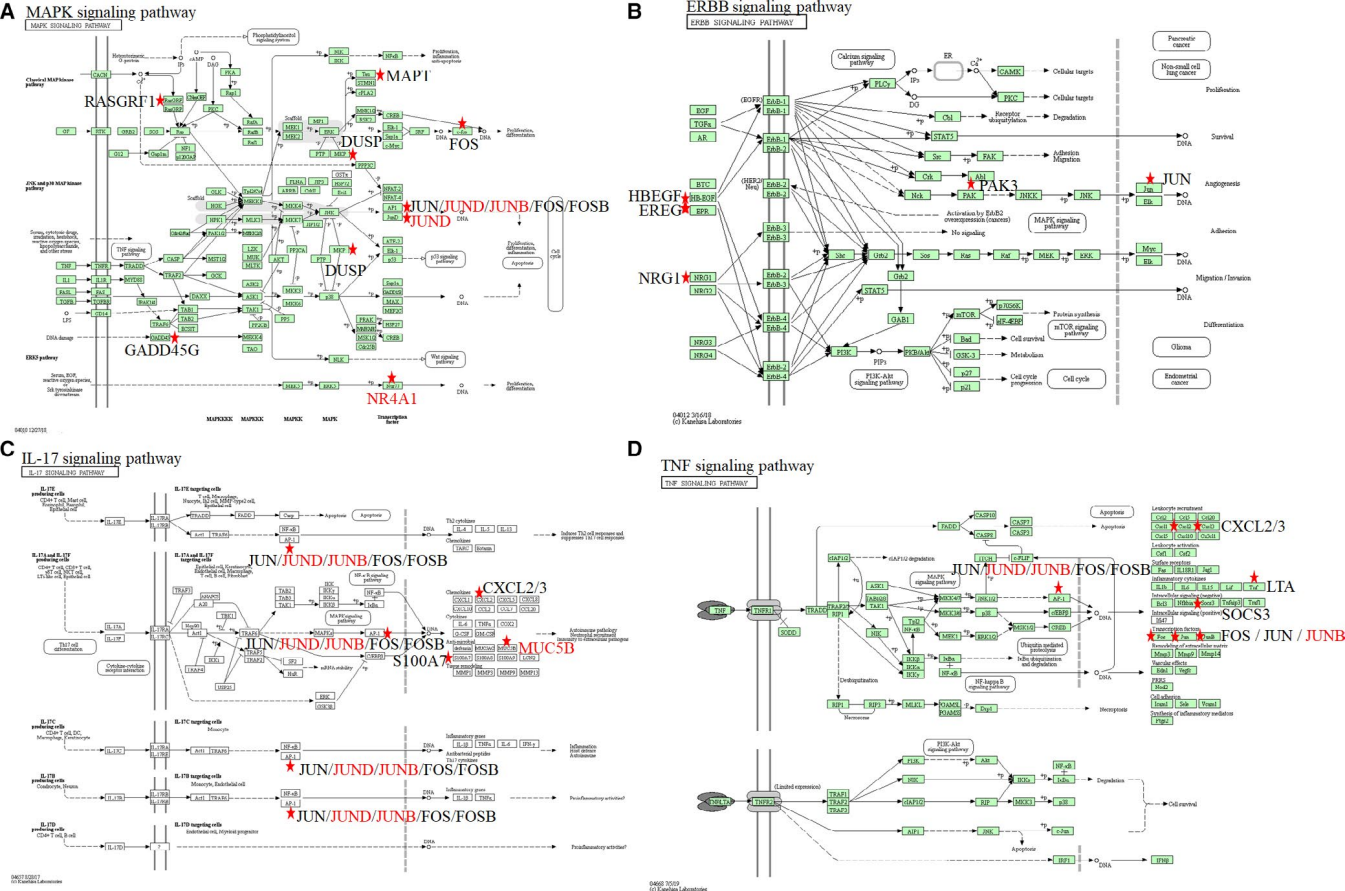
KEGG enrichment of 249 DEGs identified between chemotherapy sensitive and insensitive OC. (A) MAPK signaling pathway including RASGRF1, MAPT, FOS, FOSB, DUSP family, JUN, JUND, JUNB, NR4A1, and GADD45G. (B) ERBB signaling pathway including NRG1, EREG, HBEGF, PAK3, and JUN. (C) IL17 signaling pathway including JUN, JUND, JUNB, FOS, FOSB, S100A7, CXCL2/3, and MUC5B. (D) TNF signaling pathway, including JUN, JUND, JUNB, FOS, FOSB, CXCL2/3, LTA, and SOCS3. Common DEGs in chemotherapy sensitivity group and ovarian cancer occurring group were shown by red letter

KEGG analysis showed that DEGs were mainly enriched in four cancer‐related signaling pathways including MAPK signaling pathway (Figure 3A), ERBB signaling pathway (Figure 3B), IL‐17 signaling pathway (Figure 3C) and TNF signaling pathway (Figure 3D). MAPK and ERBB pathway were reported to be associated with cisplatin resistance in OC patients. MAPK inhibitors combined with miR‐139‐5p or metformin could improve cisplatin sensitivity in cisplatin‐resistant ovarian cancer.^24^, ^25^ NRF2 could affect the sensitivity of ovarian cancer cells to rapatinib and erotinib by regulating the ERBB signaling pathway.^26^ IL‐17 and TNF signaling pathway were identified to be related with chemotherapy sensitivity in ovarian cancer patients for the first time.

### 3.3 Key genes associated with chemotherapy sensitivity in OC patients

To identify the crucial genes that were associated with chemotherapy sensitivity in OC, we picked out 35 DEGs which were shared in both chemotherapy sensitivity group and OC occurring group (Table 3). Among them, five common DEGs (MUC5B, NR4A1, JUND, NRG1, and JUNB) participating in the four chemotherapy sensitivity‐related signaling pathways were regarded as key chemotherapy sensitive genes (Figure 3). We found that JUND, JUNB, NR4A1, and NRG1 were down‐regulated in chemotherapy sensitive OC patients while MUC5B was up‐regulated in chemotherapy sensitive OC patients.

**TABLE 3:** Common DEGs in chemotherapy sensitivity group and ovarian cancer occurring group

		Chemotherapy sensitivity vs insensitivity		Ovarian cancer vs healthy controls	
Name	ENSG	LogFC	*P* value	LogFC	*P* value
PON1	ENSG00000005421	1.833847	.008056	3.841751	.00087
GABRP	ENSG00000094755	1.879524	.009199	6.357848	.00017
TSPAN12	ENSG00000106025	−1.50372	.001892	2.66721	.000167
WNT3	ENSG00000108379	1.45233	.008192	−1.77304	.006368
CYP27B1	ENSG00000111012	−1.87876	.005665	1.905459	.003234
REEP6	ENSG00000115255	−1.44408	.000177	1.561005	.001993
**MUC5B**	ENSG00000117983	3.194437	.005183	5.930497	.000787
PAEP	ENSG00000122133	3.097212	9.58E‐05	13.58829	1.56E‐06
**NR4A1**	ENSG00000123358	−3.9535	2.23E‐11	−2.98467	.001218
SLC12A5	ENSG00000124140	1.676727	.002127	4.846811	.000172
PDE11A	ENSG00000128655	4.557034	1.80E‐07	−3.52042	9.74E‐05
UNC13A	ENSG00000130477	−1.92861	.004804	4.392345	1.26E‐08
**JUND**	ENSG00000130522	−1.06396	.000781	−1.18872	.00886
RIDA	ENSG00000132541	−1.15846	.001495	1.725161	2.37E‐06
CCNA1	ENSG00000133101	−1.8558	.00352	3.134505	.000824
ADAMTS8	ENSG00000134917	−1.58246	.006785	−2.73841	.004266
FAM129A	ENSG00000135842	1.027988	.001155	1.553927	.006426
KLF4	ENSG00000136826	−1.28913	.001771	−2.14326	.002117
CTSV	ENSG00000136943	−1.2422	.008885	5.136691	6.79E‐05
SLC38A4	ENSG00000139209	−3.13203	.00018	3.060874	.002518
ASXL3	ENSG00000141431	−2.13847	.006873	−1.64258	.004782
PRDM16	ENSG00000142611	−2.10995	.002232	−4.42514	1.04E‐08
CSRNP1	ENSG00000144655	−1.54792	2.03E‐05	−1.68077	.003687
AKAP6	ENSG00000151320	−1.70078	.000439	−2.50857	6.06E‐07
**NRG1**	ENSG00000157168	−2.76012	.001689	−3.44511	.001391
COX6B2	ENSG00000160471	1.839231	.005223	3.009415	.002123
ATF3	ENSG00000162772	−2.2823	3.72E‐05	−2.99682	.000605
**JUNB**	ENSG00000171223	−1.5749	.000159	−2.57489	.002806
APLN	ENSG00000171388	1.220806	.008744	−4.08733	1.16E‐06
PER1	ENSG00000179094	−1.28081	.000103	1.871473	.000631
EDARADD	ENSG00000186197	1.288147	.002622	3.464221	.000759
KRT16	ENSG00000186832	−2.44136	.002875	5.150993	.00058
C11orf96	ENSG00000187479	−1.95876	.000101	−2.61812	.000162
COL25A1	ENSG00000188517	−1.7561	.008887	−2.17127	.009636
PPP1R14C	ENSG00000198729	−1.44426	.009574	4.903514	.000209

Note

The bold letters represent genes involved in chemotherapy sensitive pathways.

### 3.4 Gene expression verified by qPCR and IHC

**FIGURE 4:**
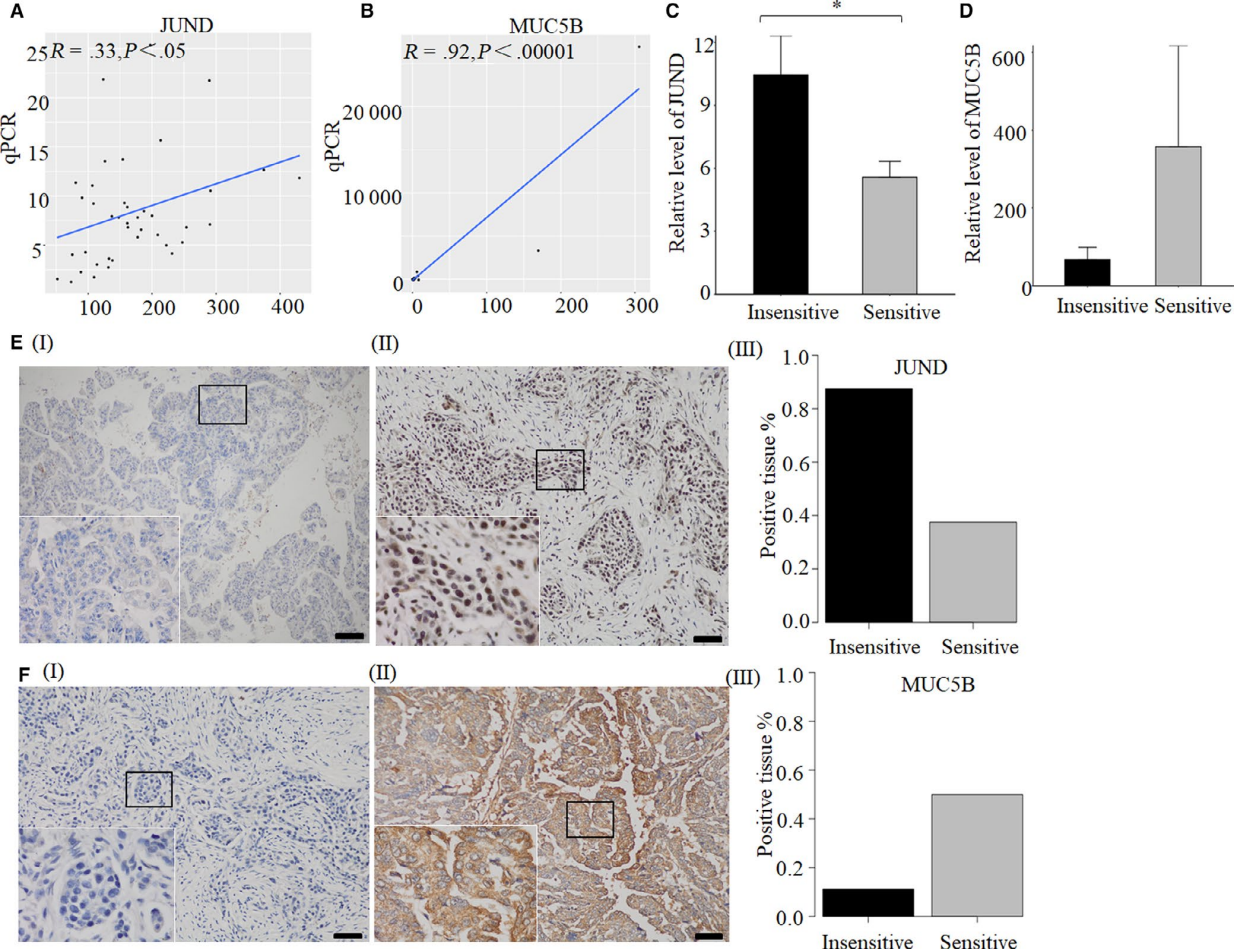
Verification of the expression levels of JUND and MUC5B in OC tissues. The correlation coefficient R between the qRCR results and TPM value of (A) JUND or (B) MUC5B in 40 or 38 OC tissues was calculated by language R, *P* < .05 was statistically significant. Comparing the relative expression levels of (C) JUND (*represent *P* < .05) and (D) MUC5B (*P* = .29) between 13 chemotherapy sensitive and 14 insensitive tissues by qPCR, β‐actin as an internal reference gene. (E) The expression of JUND in chemotherapy sensitive and insensitive tissues was tested by IHC. (I) Negative expression of JUND in chemotherapy sensitive tissues of OC. (II) Positive expression of JUND in chemotherapy insensitive tissues of OC. (III) The positive rate of JUND expressed in eight chemotherapy sensitive and eight insensitive OC tissues. (F) The expression of MUC5B in chemotherapy sensitive and insensitive tissues was tested by IHC. (I) Negative expression of MUC5B in chemotherapy insensitive tissues of OC. (II) Positive expression of MUC5B in chemotherapy sensitive tissues of OC. (III) The positive rate of MUC5B expressed in eight chemotherapy sensitive and nine insensitive OC tissues. Antibodies of JunD (Abcam, ab28837, 1:200) and MUC5B (Abcam, ab87376, 1:100) were used. The size of the ruler is 50 μm

JUND and MUC5B are involved in IL‐17 signaling pathway, and JUND is in the upstream of MUC5B. So we think these two genes are worth to be studied. Their expression levels were further verified by qPCR and IHC. The results of NGS and qPCR existed significant correlation for JUND (n = 40, *R* = .33, *P* < .05) and MUC5B (n = 38, *R* = .65, *P* < .0001) (Figure 4A,B). The relative expression level of JUND was decreased significantly in 13 sensitive tissues compared with 14 insensitive tissues (*P* < .05) (Figure 4C). While the relative level of MUC5B was increased in 13 sensitive tissues but without statistically significant (*P* = .29) (Figure 4D). IHC was performed in eight sensitive tissues and eight insensitive tissues to verify the expression level of JUND and MUC5B. The results showed that, the positive rate of JUND expression in the chemotherapy sensitive group was 37.5%, which was lower than chemotherapy insensitive group (87.5%) (Figure 4E). Fifty percent of samples were positive expression of MUC5B in chemotherapy sensitive group, while the positive rate was 11.1% in chemotherapy insensitive group (Figure 4F).

## 4 DISCUSSION

In this study, JUND, JUNB, MUC5B,^27^ NRG1^28^ and NR4A1^29^ were identified as the key genes associated with chemotherapy sensitivity in OC by NGS and bioinformatics technology. These genes are involved in four chemotherapy sensitivity‐related signaling pathways (MAPK signaling pathway,^24^, ^25^ ERBB signaling pathway,^26^ TNF signaling pathway, and IL‐17 signaling pathway). Especially, JUND and MUC5B are negative correlated in IL‐17 signaling pathway. Ju et al^27^ also showed that MUC5B was a down‐regulated gene in chemotherapy resistant epithelial ovarian cancer. Here, we further found its upstream gene JUND associated with chemotherapy for the first time. We showed that JUND was down‐regulated in chemotherapy sensitive patients. Furthermore, the differential expression of JUND in qPCR and IHC was much significant. Thus, JUND will be a good marker to predict chemotherapy effect. Our results also provide a basis for additionally functional studies that inhibiting of JUND expression may increase chemotherapy sensitivity in OC patients.

JunD and JunB are sub‐units of activator protein‐1 (AP‐1) which plays an important role in the regulation of cell proliferation, apoptosis and angiogenesis.^30^ In our study, JUNB gene was also down‐regulated in chemotherapy sensitive patients, which was consistent with JUND expression. The high expression of JUNB may predict a poor prognosis for patients with OC was also reported by Teng et al.^31^

NRG1 gene encodes Neuregulin‐1 protein, one of the ligands for members of the ErbB/epidermal growth factor‐receptor family. It was reported that down‐regulation of NRG1 expression could sensitize ovarian tumors to low cisplatin concentration.^28^ Our analysis by NGS and bioinformatics also showed that NRG1 gene was down‐regulated in chemotherapy sensitive OC patients. NR4A1 gene encodes a member of the steroid‐thyroid hormone‐retinoid receptor superfamily which acts as a nuclear transcription factor to induce apoptosis. Its role in ovarian cancer has not been determined. A study showed that NR4A1 expression was significantly lower in platinum‐resistant tumors in patients with metastatic OC, and low NR4A1 staining was associated with poorer prognosis.^29^ However, our results demonstrated that NR4A1 was a down‐regulated gene in chemotherapy sensitive OC patients. Another literature also reported that high expression of NR4A1 in high grade serous ovarian cancers had worse prognosis.^32^ The discordance in these studies about the relationship between NR4A1 and chemotherapy is perhaps induced by the limited clinical samples. We should expand samples for further study.

In summary, TNF signaling pathway and IL‐17 signaling pathway were firstly identified as OC chemotherapy sensitivity‐related pathways. JUND was firstly identified as key genes associated with chemotherapy sensitivity in OC patients. In IL‐17 signaling pathway, JUND/JUNB might transcriptional regulate MUC5B to influence the chemotherapy sensitivity in OC patients. We will further study the interaction between JUND/JUNB and MUC5B in vivo and in vitro to uncover the mechanism of chemotherapy sensitivity in ovarian cancer. More samples will be collected to verify the conclusions. Our findings provide a valuable reference for prediction of chemotherapy response in ovarian cancer patients.

## CONFLICT OF INTEREST

The authors declare that they have no competing interests.

## AUTHOR CONTRIBUTIONS

Hui Zheng contributed to study design, data acquisition and analysis, literature search, figures, and writing. Meiqin Zhang contributed to provision of study materials or patients, analysis, and interpretation of data. Shuang Ma contributed to literature search, figures, and writing. Wenting Yang contributed to data collection and analysis. Suhong Xie and Yanchun Wang contributed to technical support. Yixuan Liu, Jinyan Kai, and Qian Ma contributed to some basic experiments. Renquan Lu and Lin Guo contributed to study concept and design, administrative support, data interpretation, and article revision.

## ETHICS APPROVAL AND CONSENT TO PARTICIPATE

The research was authorized by the Ethics Committee of Fudan University Shanghai Cancer Center and informed consent was obtained from all patients.

## Data Availability

The data that support the findings of this study are available from the corresponding author upon reasonable request.
